# Supplementary material for: Rapid tuning shifts in human auditory cortex enhance speech intelligibility
Source: Nat Commun. 2016 Dec 20;7:13654. doi: 10.1038/ncomms13654 (PMC5187445; doi:10.1038/ncomms13654)
Supplement: Supplementary Information — Supplementary Figures 1 - 7, Supplementary Methods and Supplementary References [file ncomms13654-s1.pdf]

Supplementary Figures

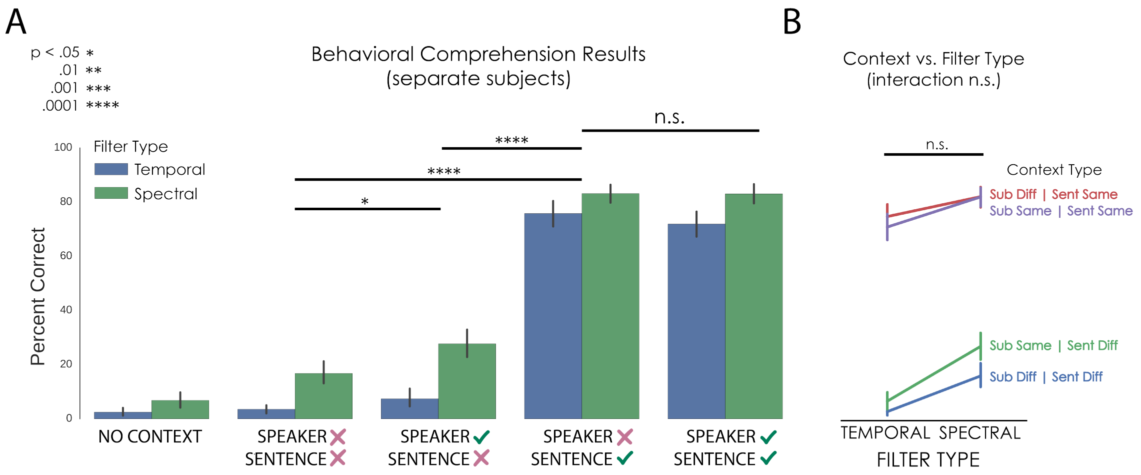

Supplementary Figure 1

Behavioral task and controls

A behavioral task was conducted to assess the effect of filter type and context type on perceptual enhancement. We ran a sentence comprehension test with several groups of undergraduate students at UC Berkeley. All participants listened to combinations of filtered speech sentences and contextual sentences, and were asked to type out all words that they understood. Comparisons were made between the percentage of words correct in each group. (A) Bar plots show speech comprehension for various combinations of high/low context. First plot: a group of subjects responded to a single presentation of a filtered speech sentence, this is denoted the “no context” condition, and serves as a baseline for comprehension (corresponding to the BEFORE condition). Remaining plots, from left to right: different sentence/different speaker (to test the effect of repetition and potentially global arousal/activation of language network caused by speech), different sentence/same speaker (voice overlap that enhances the similarity with phonemes but not

words and word order), same sentence/different speaker (phonemic and spectrally different, but with the same word identities spoken), and same sentence/same speaker (with spectral, phonemic, and word identity/rate overlap). Percentage reported correct are as follows. No Context: 4.5 +/- 0.8%. Diff Sentence, Diff Speaker: 10.4 +/- 1.2%. Diff Sentence, Same Speaker: 18.9 +/- 1.7%. Same Sentence, Different Speaker: 79.6 +/- 1.5%. Same Sentence, Same Speaker: 77.7 +/- 1.5%. Horizontal lines show significance values, and the following comparisons are all relative to the Different Speaker, Different Sentence condition: there was a main increase for Same Sentence, Different Speaker (t=9.65, df=22; showing the main perceptual enhancement effect). There is a small but significant increase of Same Speaker, Different Sentence (t=2.39, df=22; suggesting that acoustic properties of the speaker's voice is helpful in understanding the noisy stimulus). There is a much larger increase for the Same Sentence, Different Speaker condition (t=9.74, df=28; suggesting that linguistic properties of the speech are more important than acoustic properties in the speaker's voice). Finally, there is no significant difference between Same Sentence / Same Speaker and Same Sentence / Different Speaker (t=0.26, df=22; suggesting that the linguistic information shared between the two is responsible for the perceptual enhancement effect). (B) Shows the effect of filter type on perceptual enhancement in each context condition (means in each group +/- standard error). A linear mixed effects model (n observations=96 and n individuals=23) was used to calculate main effects of filter type and context on perceptual enhancement (as well as their interaction). There was a small and nonsignificant main effect of filter type (spectral > temporal, p=.082, confidence interval -41.61 to 2.47), and no significant interaction between filter type and context type, suggesting that the perceptual enhancement effect is

similar across stimulus filters (interaction term, p=.61, confidence interval -5.64 to 10.73).

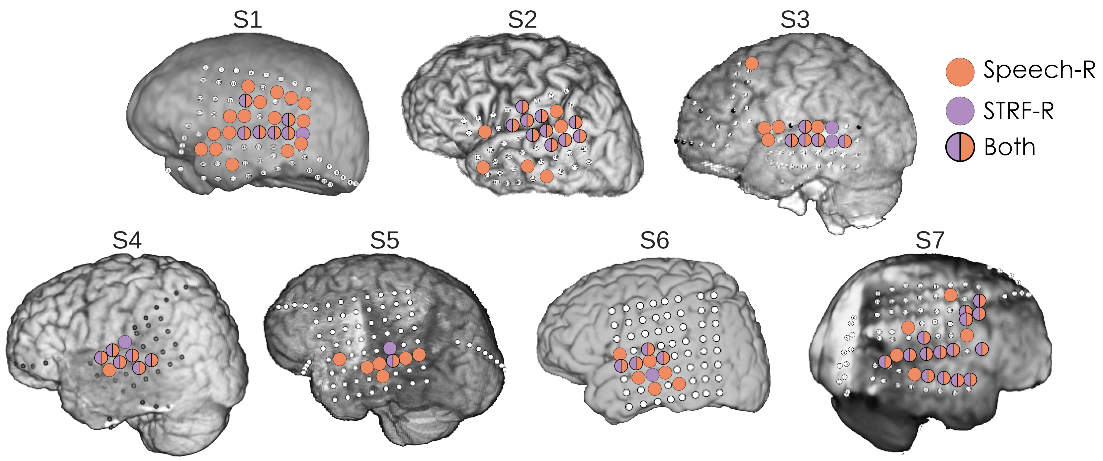

Supplementary Figure 2

Comparison of Speech-R and STRF-R electrodes

Electrodes were characterized as responsive to speech (Speech-R) if their evoked HFB activity was significantly greater than 0 (z-score over baseline, confidence interval test across trials). Electrodes were characterized as well-modeled by spectro-temporal features (STRF-R) if their goodness of fit on held-out data was greater than 0 (confidence interval test across CV splits). Anatomical distribution of Speech-R electrodes (orange), STRF-R electrodes (purple) and electrodes responsive to both (split colors) are shown above.

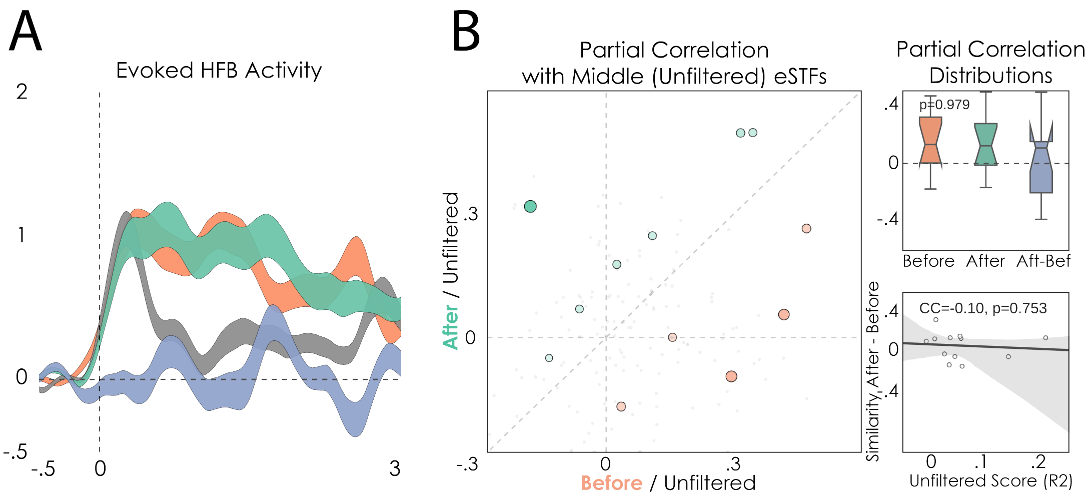

Supplementary Figure 3

Analysis during pink noise trials

A subset of subjects (n=3) performed a pink noise control on half of their trials. Instead of an unfiltered speech context in the MIDDLE condition, energy-matched pink noise was played. (A) HFB activity (mean +/- standard error across all electrodes included in the analysis) is shown. The difference (AFTER - BEFORE) is plotted in purple. See Figure 3 for details. (B) eSTRFs were calculated for electrodes during pink-noise conditions. The similarity between BEFORE/MIDDLE, and AFTER/MIDDLE eSTRFs was estimated using partial correlation coefficients (see methods and text) and these are shown in the middle scatterplot. There was no significant difference in partial correlation for BEFORE/MIDDLE vs. AFTER/MIDDLE conditions (permutation test, n=12). See Figure 10 for additional details.

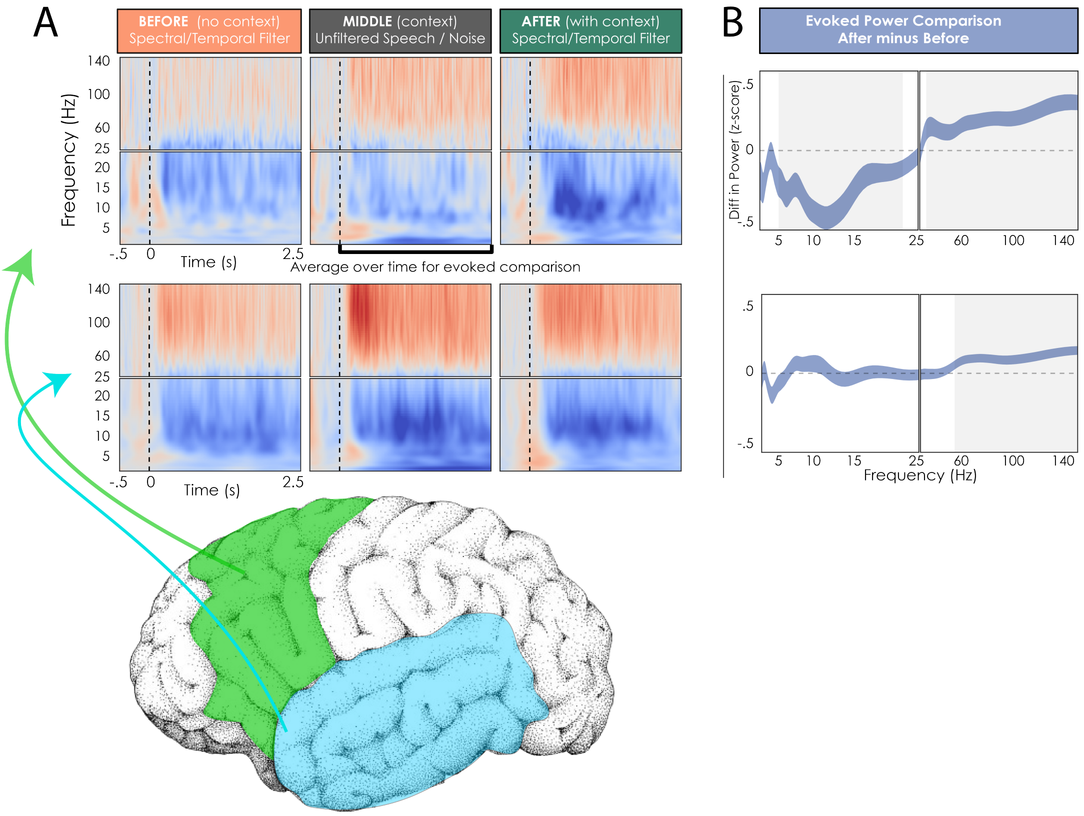

Supplementary Figure 4

Time Frequency Response and Frequency Band Selection

For each electrode, the ECoG signal was convolved with 100 log-spaced Morlet wavelets (number of cycles fixed at 5) to create a time-varying power in each frequency band. Power was averaged within anatomical region and compared across conditions. (A) Mean TFR for Frontal (Top) and Temporal (Bottom) electrodes across conditions. (B) Evoked TFR in post-stimulus time points was averaged for frequency band selection. The mean +/- standard error is shown for each frequency. A cluster-based permutation test was used to find frequency-specific differences in power between the BEFORE and AFTER condition. Power in the high-frequency broadband (HFB) range significantly increased in the AFTER condition in both frontal and temporal electrodes (frontal, p=.001, n=75;

temporal, p=.002, n=217). There was also a decrease in frequencies below 25 Hz in frontal electrodes (p=.001, n=75). This prompted further investigation of the HFB signal.

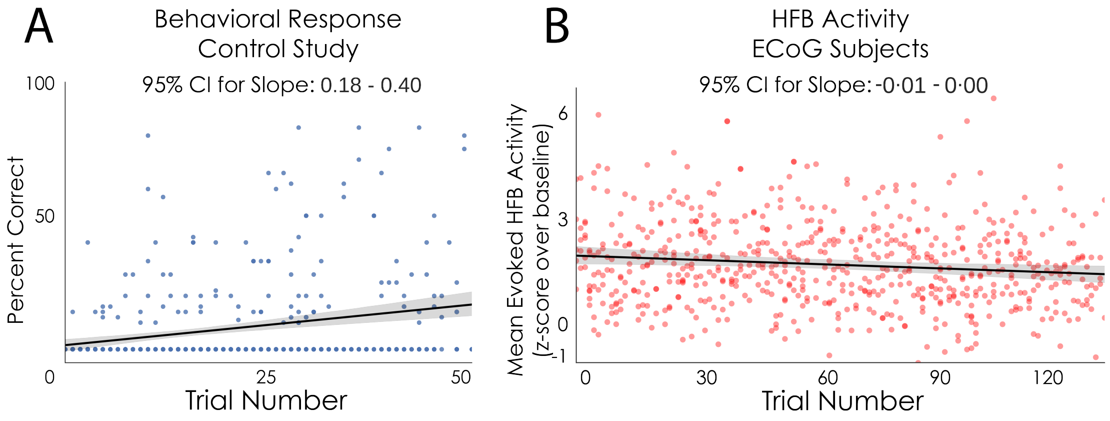

Supplementary Figure 5

Behavioral and HFB change over trials

(A) In behavioral control subjects, the percent correct words are plotted as a function of trial number for subjects that heard either no context, or a Different Subject, Different Sentence context (testing the effect of repeated exposure to the filtered speech stimuli, n=14). We used a bootstrap technique to calculate confidence intervals on the slope of the line relating the percent words correct to the trial number. Bootstrapped regression coefficients found a slightly positive relationship between percent correct and trial number, suggesting a small effect of session duration on perceptual enhancement. (B) The same bootstrapped regression approach was applied to mean HFB activity in ECoG subjects, using electrodes that showed an increase in HFB activity to speech (Speech-R). Bootstrapped coefficients show a non-significant relationship between HFB activity and trial number.

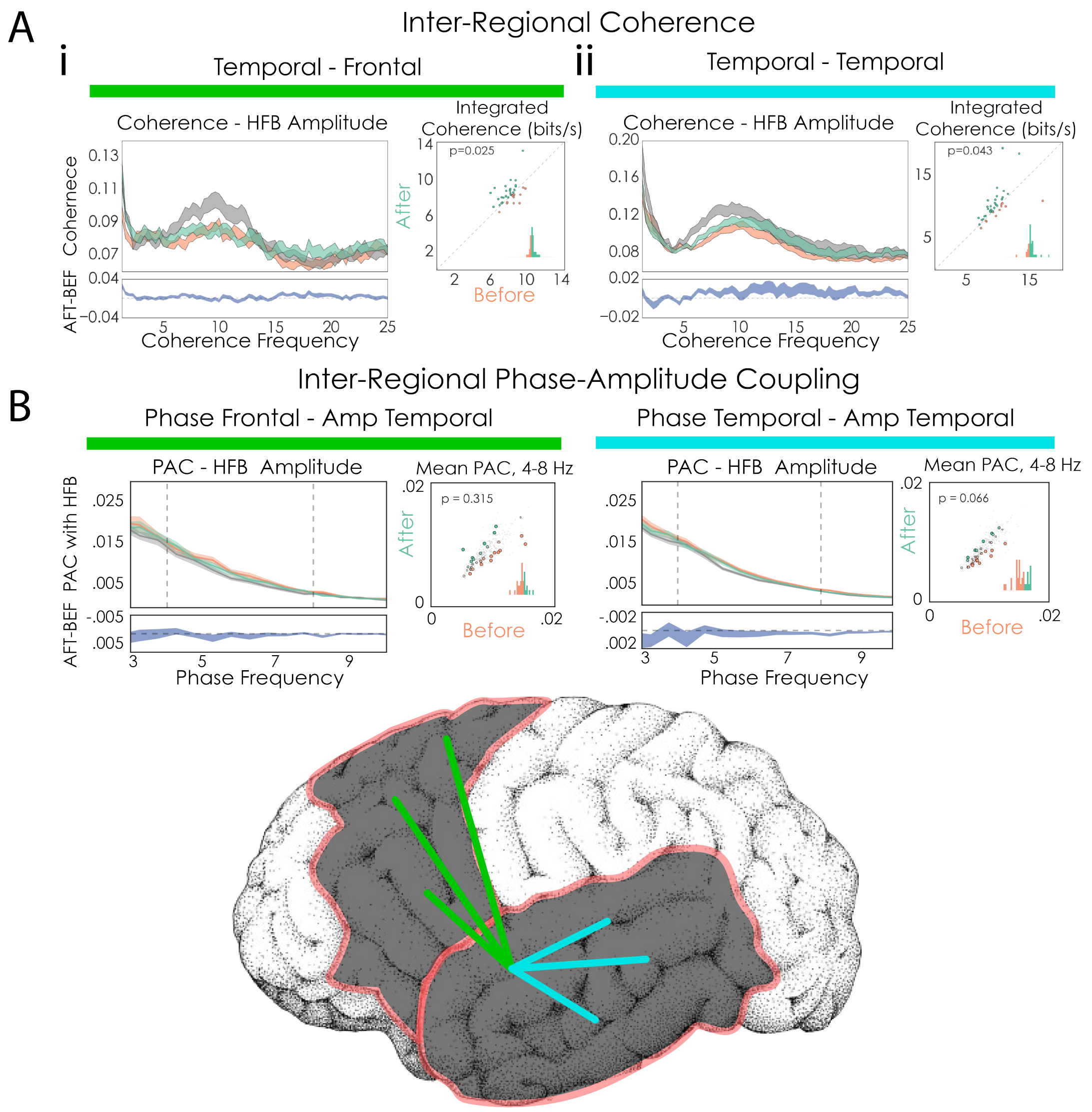

Supplementary Figure 6

Connectivity Analyses

(A) Coherence was calculated between electrodes in regions of interest. Mean +/- standard error are shown across electrodes. We defined a set of "seed" electrodes in the temporal lobe that were Speech-R and STRF-R. For each seed, the coherence in the HFB amplitude was calculated between it and all other electrodes in the frontal (left, i) or temporal (right, ii) lobe. For each seed electrode, coherence values were averaged across

targets and, converted into bits/second, and integrated across frequencies. These values are plotted in the scatterplots to the right along with p-values for the difference between AFTER and BEFORE (paired permutation test, n=39). We measured a small but significant increase in coherence for both temporal-frontal electrodes (p=.025), and temporal-temporal electrodes (p=.043). (B) Phase-Amplitude Coupling was calculated the regions described in A. The time-varying phase of theta-range frequencies (2 - 12Hz, .5Hz spacing) along with the mean amplitude of HFB frequencies (70 - 140Hz, 10Hz spacing) was calculated using band-pass filters followed by a Hilbert transform (and averaging frequency bands together in the case of the high-frequency amplitude). The strength of coupling was calculated using the Phase Amplitude Coupling measure defined in Ozkurt and Schnitzler, 2011 (see supplemental methods). The large line plots on the left show the mean +/- standard error PAC as a function of the frequency of the phase in the theta-range region. The smaller line plots below show the mean +/- standard error of the difference in PAC (AFTER - BEFORE). The scatterplots on the right show the mean PAC for the 4-8Hz phase in each condition. There was no significant change in PAC between frontal and temporal electrodes in the AFTER condition relative to the BEFORE condition (paired permutation t-test).

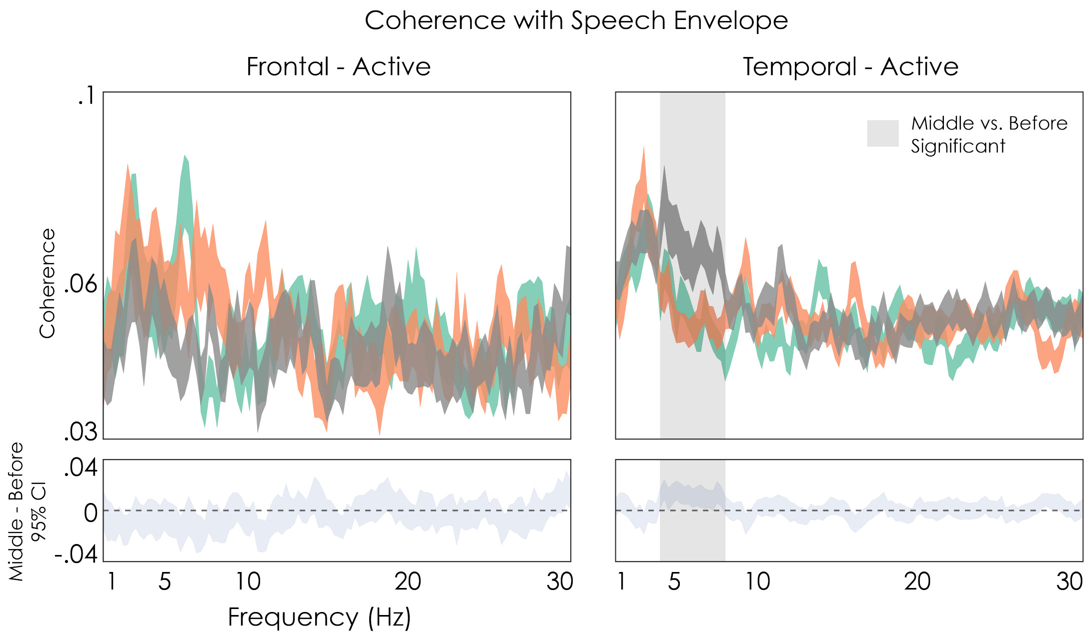

Supplementary Figure 7

Coherence with Speech Envelope

Since prior studies have shown that low frequencies in the ECoG signal tracked the speech envelope and that this tracking was modulated by attentional processes[1], we also examined whether we could detect a similar effect in our study. The envelope of speech in each condition was calculated in each condition by averaging across power from 64 wavelets log-spaced from frequencies 500 to 2000Hz. Post-stimulus coherence (0s to 3s) between the raw ECoG signal and the speech envelope was calculated for all electrodes with HFB activity (Speech-R). Top row: mean +/- standard error coherence is plotted for frontal (left) and temporal (right) electrodes. Bottom row: the difference in condition (MIDDLE - BEFORE) is plotted (mean +/- standard error) across active electrodes. In the temporal lobe, there was a significant increase in theta coherence during unfiltered speech relative to the BEFORE condition (permutation cluster test, p=.001, n=72), but no significant difference between BEFORE and AFTER conditions. There were no

significant effects in frontal electrodes. Other neurophysiological studies have correlated the signals detected in the lower frequencies of ECoG, MEG, or EEG with the envelope of human speech[2]. While our study confirms that the neural encoding of speech features changes with intelligibility, we found no change in coherence between theta activity and the speech envelope. This may be due to the different spectrotemporal properties of our filtered speech stimuli, and the fact that ECoG records signal from a different distribution of neural sources than MEG. It should be noted that these effects have generally been described in premotor/frontal regions, which did not have extensive electrode coverage in this study.

Supplementary Methods

Connectivity between frontal/temporal electrodes

To assess putative top-down signals underlying the reported eSTRF plasticity, we conducted several connectivity analyses between frontal/premotor cortex and temporal cortex. We tested whether there was an increase in frontal/temporal coherence in the AFTER condition, whether delta-theta activity in the frontal and temporal lobes was phase-locked to the speech envelope, and whether directional Phase-Amplitude Coupling between delta-theta and HFB activity was increased in the AFTER condition. Connectivity results did not yield any conclusive findings. As electrode coverage for this study was based on temporal lobe coverage, not frontal/motor coverage, this data is not well suited for answering questions about intra-cortical connectivity and how it pertains to eSTRF plasticity. Further details for these connectivity analysis and results are found below.

Inter- and Intra-regional Coherence Analysis

To investigate putative higher-level regions that may be involved in eSTRF plasticity, we conducted connectivity analyses between anatomical regions of interest. Analyses were conducted with the HFB activity of electrodes included in eSTRF analysis as seeds (Speech-R and STRF-R, located on the temporal lobe, hereafter called Temporal seeds). We performed a separate analysis for two groups of target electrodes: all other temporal lobe electrodes (Temporal targets), and electrodes located on frontal/premotor regions (hereafter, Frontal targets). It should be noted that there was generally sparse electrode

coverage in frontal/premotor regions, as grid cases were primarily selected for temporal lobe coverage.

To calculate the coherence between electrodes, we used a multi-taper windowing method similar to that described above. For each trial, the coherence was calculated between pairs of electrodes with seeds/targets based on anatomical regions of interest (and only including electrodes included in the eSTRF analysis as seeds). For each seed, coherence was averaged across target electrodes. Next, the mean coherence was converted to normal mutual information by integrating across frequencies. The difference in condition (AFTER – BEFORE) was calculated for each electrode, and a permutation t-test was conducted across electrodes to test for a difference in condition. Coherence calculation was performed with the MNE-toolbox (see Data Availability in the main manuscript for information about how to access this code).

The coherence was first calculated between Temporal seeds and Frontal targets. The results are shown in Supplementary Figure 6A. There was a frequency peak around 9-10Hz, and a weak general increase in coherence in the AFTER condition over the BEFORE condition. For Temporal seeds and Temporal targets, there was a broadband increase in coherence across frequencies greater than 5Hz. (permutation test, see Supplementary Figure 6A for discussion).

Theta Connectivity Phase-Amplitude Coupling Analysis

Next, we investigated the role of the theta band in modulating HFB activity. It has been suggested that the phase of theta activity may modulate the amplitude of HFB activity, representing a neural mechanism by which associative cortical areas influence the

processing occurring in sensory cortex[3,4]. We calculated the Phase-Amplitude Coupling (PAC) between electrodes included in the eSTRF analysis and groups of electrodes either in temporal or frontal/pre-motor regions.

To test for cross-frequency effects between theta phase and HFB amplitude, we split electrodes into groups to be analyzed for phase and for amplitude. To calculate phase, we band-pass filtered the signal from each electrode, then calculated the time-varying phase of the Hilbert transform of each of the band-limited signals. We calculated the phase for frequencies from 3Hz to 10Hz in increments of 0.5Hz. To calculate the HFB amplitude of each signal, we again performed a band-pass filter of the raw signal for 10 logarithmically-spaced bands from 70-140Hz. For each band we calculated the modulus of the Hilbert transform and averaged the bands together.

We calculated the Phase-Amplitude Coupling using the method described in Ozkurt and Schnitzler, 2011. This is form of the Modulation Index that is normalized by the amplitudes of the two filtered signals[5]. For each condition, we concatenated the phases/amplitudes of the pair of electrodes across trials, then calculated a single value for PAC between electrodes. To test for a difference in condition (AFTER – BEFORE), we took the average PAC for phases from 3-8Hz, and the amplitude from the HFB signal, and calculated the difference AFTER – BEFORE. For each HFB amplitude electrode, we averaged the PAC value across all other theta phase electrodes to calculate a single value of PAC for each HFB amplitude electrode. Significance for the difference in condition (AFTER – BEFORE) was assessed with a permutation t-test for a difference from 0 (see Data Availability in the main manuscript for information about how to access this code).

As previously mentioned this study did not include dense coverage over premotor/frontal regions, and the reported effect sizes are small, precluding a conclusive result. Future studies should investigate the putative link between frontal and temporal electrodes in top-down mechanisms of speech perception.

Electrode coherence with speech envelope

There have been several studies suggesting a role of theta band activity in parsing speech utterances, especially in noise[2,6]. Previous studies have suggested that coherence between neural activity and the speech envelope increases in the theta band during intelligible speech. This entrainment has been interpreted as the tracking of the rhythmic structure of an attended speech stimulus by associative cortex, which facilitates speech processing. To investigate whether these effects are modulated by experience with intact speech, we calculated the coherence between the raw electrode signal and the speech envelope.

For each trial, we calculated the envelope of the speech stimulus by first performing a time-frequency decomposition of the audio waveform using 64 log-spaced frequency Gabor wavelets with center frequencies from 500 to 2000Hz. The 64 band-passed signals were rectified and then averaged across frequencies.

To calculate the coherence between ECoG activity and the envelope of each speech stimulus, we again used a multi-taper windowing method. For each trial, we calculated the coherence between electrodes of interest and the speech envelope. We converted this value to normal mutual information. To find particular frequencies that showed a difference in electrode-envelope coherence between conditions, we calculated the difference between conditions for each frequency band. We tested the difference in

condition (AFTER – BEFORE, and MIDDLE – BEFORE) by conducting a cluster-based permutation t-test for a difference from 0 (see Supplementary Figure 7).

Supplementary References

1. Zion Golumbic, E. M. *et al.* Mechanisms Underlying Selective Neuronal Tracking of Attended Speech at a 'Cocktail Party'. *Neuron* **77,** 980–991 (2013).
2. Peelle, J. E., Gross, J. & Davis, M. H. Phase-locked responses to speech in human auditory cortex are enhanced during comprehension. *Cereb. Cortex* **23,** 1378–87 (2013).
3. Canolty, R. T. *et al.* High Gamma Power Is Phase-Locked to Theta Oscillations in Human Neocortex. *Science (80-.).* **313,** 1626–1628 (2006).
4. Voytek, B. *et al.* Oscillatory dynamics coordinating human frontal networks in support of goal maintenance. *Nat. Neurosci.* 1–10 (2015). doi:10.1038/nn.4071
5. Ozkurt, T. E. & Schnitzler, A. A critical note on the definition of phase-amplitude cross-frequency coupling. *J. Neurosci. Methods* **201,** 438–443 (2011).
6. Ding, N. & Simon, J. Z. Cortical entrainment to continuous speech: functional roles and interpretations. *Front. Hum. Neurosci.* **8,** 311 (2014).
